# Impact of effective regurgitant orifice area on outcome of secondary mitral regurgitation transcatheter repair

**DOI:** 10.1007/s00392-021-01807-0

**Published:** 2021-03-04

**Authors:** Nicole Karam, Mathias Orban, Daniel Kalbacher, Christian Butter, Fabien Praz, Edith Lubos, Marwin Bannehr, Mohammad Kassar, Aniela Petrescu, Christos Iliadis, Matthias Unterhuber, Anouk Asselin, Holger Thiele, Roman Pfister, Stephan Windecker, Philipp Lurz, Stephan von Bardeleben, Jörg Hausleiter, for the EuroSMR investigators, Mathias Orban, Daniel Braun, Michael Näbauer, Stephan Massberg, Joerg Hausleiter, Daniel Kalbacher, Edith Lubos, Dirk Westermann, Niklas Schofer, Sebastian Ludwig, Christian Butter, Marvin Bannehr, Fabien Praz, Stephan Windecker, Mohammad Kassar, Aniela Petrescu, Stephan von Bardeleben, Roman Pfister, Christos Iliadis, Stephan Baldus, Philipp Lurz, Holger Thiele, Matthias Unterhuber, Maximilian von Roeder, Thilo Noack, Nicole Karam, Tania Puscas, Alain Berrebi, Noemie Tence, Christian Latremouille

**Affiliations:** 1 Department of Cardiology and Paris Cardiovascular Research Center (INSERM U970), European Hospital Georges Pompidou, Paris, France; 2 Medizinische Klinik und Poliklinik I, Ludwig Maximilians Universität München, Munich, Germany; 3 Partner Site German Center for Cardiovascular Disease (DZHK), Munich Heart Alliance, Munich, Germany; 4 Klinik für Kardiologie, UKE Hamburg, Universitäres Herz- und Gefäßzentrum, Hamburg, Germany; 5 Department of Cardiology, Heart Center Brandenburg and Brandenburg Medical School, Bernau, Germany; 6 Department of Cardiology, University Hospital, University of Bern, Bern, Switzerland; 7 Mainz University Hospital, University of Mainz, Mainz, Germany; 8 Department III of Internal Medicine, Heart Center, University of Cologne, Faculty of Medicine and University Hospital Cologne, Cologne, Germany; 9 Department of Cardiology, Heart Center Leipzig at University of Leipzig, Leipzig, Germany

**Keywords:** Mitral regurgitation, Effective regurgitant orifice area, Edge-to-edge repair, Outcome, Heart failure with reduced ejection fraction

## Abstract

**Objectives:**

To assess the value of effective regurgitant orifice (ERO) in predicting outcome after edge-to-edge transcatheter mitral valve repair (TMVR) for secondary mitral regurgitation (SMR) and identify the optimal cut-off for patients’ selection.

**Methods:**

Using the EuroSMR (European Registry of Transcatheter Repair for Secondary Mitral Regurgitation) registry, that included patients undergoing edge-to-edge TMVR for SMR between November 2008 and January 2019 in 8 experienced European centres, we assessed the optimal ERO threshold associated with mortality in SMR patients undergoing TMVR, and compared characteristics and outcomes of patients according to baseline ERO.

**Results:**

Among 1062 patients with severe SMR and ERO quantification by proximal isovelocity surface area method in the registry, ERO was < 0.3 cm^2^ in 575 patients (54.1%), who were more symptomatic at baseline (NYHA class ≥ III: 91.4% vs. 86.9%, for ERO < vs. ≥ 0.3 cm^2^; *P* = 0.004). There was no difference in all-cause mortality at 2-year follow-up according to baseline ERO (28.3% vs. 30.0% for ERO < vs. ≥ 0.3 cm^2^, *P* = 0.585). Both patient groups demonstrated significant improvement of at least one NYHA class (61.7% and 73.8%, *P* = 0.002), resulting in a prevalence of NYHA class ≤ II at 1-year follow-up of 60.0% and 67.4% for ERO < vs. ≥ 0.3 cm^2^, respectively (*P* = 0.05).

**Conclusion:**

All-cause mortality at 2 years after TMVR does not differ if baseline ERO is < or ≥ 0.3 cm^2^, and both groups exhibit relevant clinical improvements. Accordingly, TMVR should not be withheld from patients with ERO < 0.3 cm^2^ who remain symptomatic despite optimal medical treatment, if TMVR appropriateness was determined by experienced teams in dedicated valve centres.

**Supplementary Information:**

The online version contains supplementary material available at 10.1007/s00392-021-01807-0.

## Introduction

Chronic secondary mitral regurgitation (SMR) is a frequent finding in the setting of heart failure with reduced ejection fraction (HFrEF), and is associated with adverse prognosis. Since SMR is only one component of the left heart disease, mitral valve treatment may not be by itself curative, and the best therapy for chronic SMR is unclear. The benefit of mitral surgery in this setting has not been clearly demonstrated, and the majority of these patients have multiple complex comorbidities and are denied surgery in clinical practice [1, 2].

Edge-to-edge transcatheter mitral valve repair (TMVR) has been shown to be an effective and safe therapeutic option in patients with primary MR at high surgical risk [3]. In SMR, results from real-life registries and three retrospective studies have shown promising initial results, including a potential mortality benefit over isolated optimal medical treatment (OMT) [4–8]. Recently, two prospective randomized trials assessed the benefit of TMVR on top of OMT in patients with severe SMR: the French Percutaneous Repair with the MitraClip Device for Severe Functional/Secondary Mitral Regurgitation (MITRA‐FR) trial, which showed no benefit from TMVR on top of OMT [9], and the American Cardiovascular Outcomes Assessment of the MitraClip Percutaneous Therapy for Heart Failure Patients with Functional Mitral Regurgitation (COAPT) trial, which demonstrated significant reductions in mortality and hospitalisations for heart failure as well as improvements in quality of life [10, 10]. One of the potential explanations that were discussed for the observed discrepancies in outcomes is the difference in effective regurgitant orifice (ERO) in each study: mean baseline ERO was 0.31 cm^2^ in MITRA‐FR, vs. 0.41 cm^2^ in COAPT cm^2^, which led to a questioning of the yield of TMVR in the setting of low ERO (mainly < 0.3 cm^2^), even though guidelines advocate for an integrative approach to assess the severity of MR.

Accordingly, the aim of this study was two-fold: (1) to compare the characteristics and outcomes of SMR patients undergoing edge-to-edge TMVR in the retrospective EuroSMR (European Registry of Transcatheter Repair for Secondary Mitral Regurgitation) registry, according to the ERO threshold, and (2) to assess the yield of ERO in selecting candidates for TMVR, and eventually identify an optimal ERO threshold associated with mortality in SMR patients undergoing TMVR.

## Methods

### Patients’ cohort

The EuroSMR registry is a large, retrospective registry including patients undergoing TMVR for SMR using the MitraClip^®^ device (Abbott Structural Heart, Santa Clara, CA, USA) between November 2008 and January 2019 in 8 experienced European centres (Munich, Hamburg, Berlin-Bernau, Bern, Mainz, Cologne, Leipzig, and Paris-Pompidou). All patients were symptomatic despite OMT, defined as maximally tolerated guideline-directed medical therapy, with cardiac resynchronization therapy when indicated. A heart team consensus was necessary before TMVR to evaluate the best treatment option for the individual patient. One main operator, with a secondary operator, performed the procedures in most centres, leading to a number of operators of less than 16. The study was registered at Deutsches Register Klinischer Studien (DRKS00017428).

Patient and Public Involvement: patients were not involved in the design and conduction of the study.

### Technical aspects

The procedural steps of TMVR using MitraClip^®^ have been previously described [12, 13]. In brief, the procedure is performed under general anaesthesia with fluoroscopic and transoesophageal echocardiographic guidance. The transcatheter edge-to-edge repair system is introduced via the femoral vein, and advanced to the left atrium through a transseptal puncture. The device is then aligned perpendicular to the mitral valve plane, with the MitraClip arms perpendicular to the line of coaptation. The mitral leaflets are grasped and the device is closed, resulting in fixed approximation of the mitral leaflets. One or several clips might be needed to achieve optimal result.

### Data collection

Collected data included demographic data (age and sex), medical history, and echocardiographic data. All echocardiograms were performed and analysed by experienced operators at each site. All patients underwent transthoracic and transoesophageal echocardiography before TMVR. Baseline evaluation of MR severity was performed using an integrative approach including quantitative [ERO and regurgitant volume by proximal isovelocity surface area (PISA) method] and semiquantitative parameters (vena contracta width averaged in long-axis and commissural views) as well as an assessment of the left ventricle using left ventricular ejection fraction (LVEF) and volumes (Simpson’s biplane summation of disc technique). The PISA radius was measured as the distance from the threshold of color Doppler aliasing (abrupt change in color by shifting the baseline of color scale in the direction of the regurgitant jet) to the jet orifice (Supplementary Fig. 1). MR was considered severe according to these combined criteria in all patients included in the registry. Symptomatic status was assessed using the New York Heart Association (NYHA) functional class. Patients had regular follow-ups in their respective hospitals. Available follow-up data included survival status, NYHA functional class at 1 year and 2 years, and evaluation of MR grade through either outpatient visit or telephone interviews with the patient or patient’s relatives.

Data collection and follow-ups were performed according to protocols of the participating centres, and in line with local ethical regulations and adhered to international rules for scientific studies as well as the Helsinki principles. Patients needed to be legal of age and able to consent. The authors had full access to data and designed the statistical analysis, had final responsibility for the decision to submit the manuscript for publication, and vouch for the accuracy and completeness of the data and the analyses.

### Statistical analysis

Spline curves, assessing the relation between ERO and mortality, were performed in order to identify the optimal cut-off for ERO. In case of absence of cut-off, the median ERO was intended to be used as a cut-off for the analyses. Supplementary analyses were pre-specified, using the cut-offs of 0.2 and 0.4 cm^2^, according to current guidelines.

Patients’ characteristics and outcomes were described according to their baseline ERO. The primary outcome was survival rate at 2 years. NYHA functional class was assessed and compared between the two ERO groups at baseline and last follow-up. Variables were described using medians [and interquartile ranges (IQR)] as well as means and standard deviations for continuous measures, and counts and proportions for categorical measures. Normality of presented variables was assessed using the Shapiro test and graphical distribution. Comparisons between groups were performed using Chi-square for categorical variables, and Student’s t-test or Mann–Whitney-Wilcoxon test for continuous variables, as appropriate. Cumulative survivals at two year were analysed using Kaplan–Meier models and compared using Cox regression. The cumulative incidence of the secondary end-point was compared between the two ERO groups using a Cox regression model. All *P* values were calculated using 2-sided tests, and a significance level of 0.05 was used to declare statistical significance. Statistical analyses were performed using R software version 3.5.1 (R Project for Statistical Computing). This study is reported according to Strengthening The Reporting of Observational studies in Epidemiology guidelines [14].

## Results

A total of 1237 patients with severe SMR underwent TMVR in the participating centres and were included in EuroSMR. In 1062 patients, ERO quantification before TMVR was available, which defined the study population for the current analysis. Baseline characteristics of the global population are presented in Table 1. Median age of the patients was 76.0 (IQR: 69.0–80.1) years, with 387 women (36.5%). Overall, 943 patients (89.4%) were in NYHA class III or IV at the time of the procedure. They presented with atrial fibrillation in 686 patients (64.7%), a prior stroke in 98 patients (9.2%), and a chronic pulmonary disease in 174 patients (16.4%). Regarding surgical risk, median EuroSCORE II was 6.7% (IQR: 3.9–11.9%). The aetiology of SMR was ischemic in 515 patients (51.6%).

**Table 1:** Baseline clinical characteristics according to ERO

	Overall 1062 patients	ERO < 0.3 575 patients	ERO ≥ 0.3 487 patients	P*
Age, years	76.0 [69.0–80.1]	76.0 [69.0–80.2]	75.0 [69.0–80.1]	0.109
Male sex, *n* (%)	673 (63.5%)	348 (60.7%)	325 (66.7%)	0.050
EuroSCORE II, %	6.7 [3.9–11.9]	6.6 [3.9–11.6]	6.9 [3.9–12.5]	0.521
Ischemic aetiology, *n* (%)	515 (51.6%)	290 (54.9%)	225 (47.9%)	0.031
Prior CABG, *n* (%)	174 (17.5%)	98 (18.8%)	76 (16.1%)	0.299
Previous PCI, *n* (%)	346 (42.8%)	182 (44.9%)	164 (40.7%)	0.251
Prior myocardial infarction, *n* (%)	261 (24.9%)	139 (24.5%)	122 (25.4%)	0.806
Prior stroke, *n* (%)	98 (9.2%)	53 (9.2%)	45 (9.2%)	0.999
Chronic Pulmonary Disease, *n* (%)	174 (16.4%)	98 (17.1%)	76 (15.7%)	0.587
Atrial fibrillation, *n* (%)	469 (62.9%)	274 (61.9%)	195 (64.4%)	0.573
ICD, *n* (%)	179 (24.9%)	91 (26.1%)	88 (23.8%)	0.547
CRT, *n* (%)	162 (19.0%)	79 (18.6%)	83 (19.4%)	0.845
GFR, (ml/min)	46.1 [33.0—61.1]	46.0 [33.0—63.1]	46.2 [33.0—60.0]	0.730
NYHA class III or IV, *n* (%)	943 (89.4%)	524 (91.4%)	419 (86.9%)	0.004

Qualitative data are presented as *n* (%); quantitative data are presented as mean ± SD. *CABG* coronary artery bypass graft, *PCI* percutaneous Coronary Intervention, ICD implantable cardioverter-defibrillator, *CRT* cardiac resynchronization therapy, *GFR* glomerular filtration rate, *NYHA* New York Heart Association

*Parametric tests for continuous normal variables, non-parametric tests for continuous non-normal variables, *X*^2^ for categorical variables

Baseline echocardiographic parameters are described in Table 2. Median ERO was 0.28 cm^2^ (IQR: 0.20–0.39). Median LVEF was 34.0% (IQR: 25.0–44.0%) with a median LV end-diastolic volume of 170 ml (IQR: 123–225 ml) and end-systolic volume of 110 ml (IQR: 70.0–160.0 ml). The median vena contracta diameter was 6.8 mm (IQR: 4.8–8.0 mm).

**Table 2:** Baseline clinical echocardiographic according to ERO

	Overall 1062 patients	ERO < 0.3 575 patients	ERO ≥ 0.3 487 patients	P*
ERO (PISA), cm^2^	0.28 [0.20–0.39]	0.20 [0.15–0.24]	0.40 [0.33–0.49]	< 0.001
RV (PISA), ml	36.0 [25.0;50.0]	28.0 [21.6;36.0]	52.0 [43.5;67.0]	< 0.001
Vena contracta, mm	6.8 [4.8–8.0]	6.5 [4.4–7.8]	7.0 [5.0–8.1]	0.018
MV characteristics				
Mean pressure gradient, mmHg	1.00 [1.00;2.00]	1.00 [1.00;2.00]	2.00 [1.00;2.00]	0.534
Antero-posterior diameter, cm	3.62 [3.27;4.04]	3.56 [3.22;4.00]	3.80 [3.39;4.14]	0.020
Tenting height, cm	0.78 [0.60;0.98]	0.75 [0.57;0.92]	0.88 [0.70;1.03]	0.002
Tenting area, cm^2^	2.78 [2.14;3.41]	2.70 [2.10;3.33]	2.90 [2.33;3.89]	0.029
LV characteristics				
LVEF, %	34.0 [25.0–44.0]	34.0 [25.8–44.7]	33.2 [25.0–43.0]	0.415
End-diastolic volume, ml	170 [123–225]	166 [118–215]	174 [130–240]	0.002
End-systolic volume, ml	110 [70–160]	108 [68–154]	114 [74–168]	0.061

*Parametric tests for continuous normal variables, non-parametric tests for continuous non-normal variables, *X*^2^ for categorical variables. *ERO* effective regurgitant orifice, *PISA* proximal isovelocity surface area, *RV* regurgitant volume, *MV* mitral valve, *LV* left ventricular, *LVEF* left ventricular ejection fraction

### Characteristics according to ERO

**Fig. 1:**
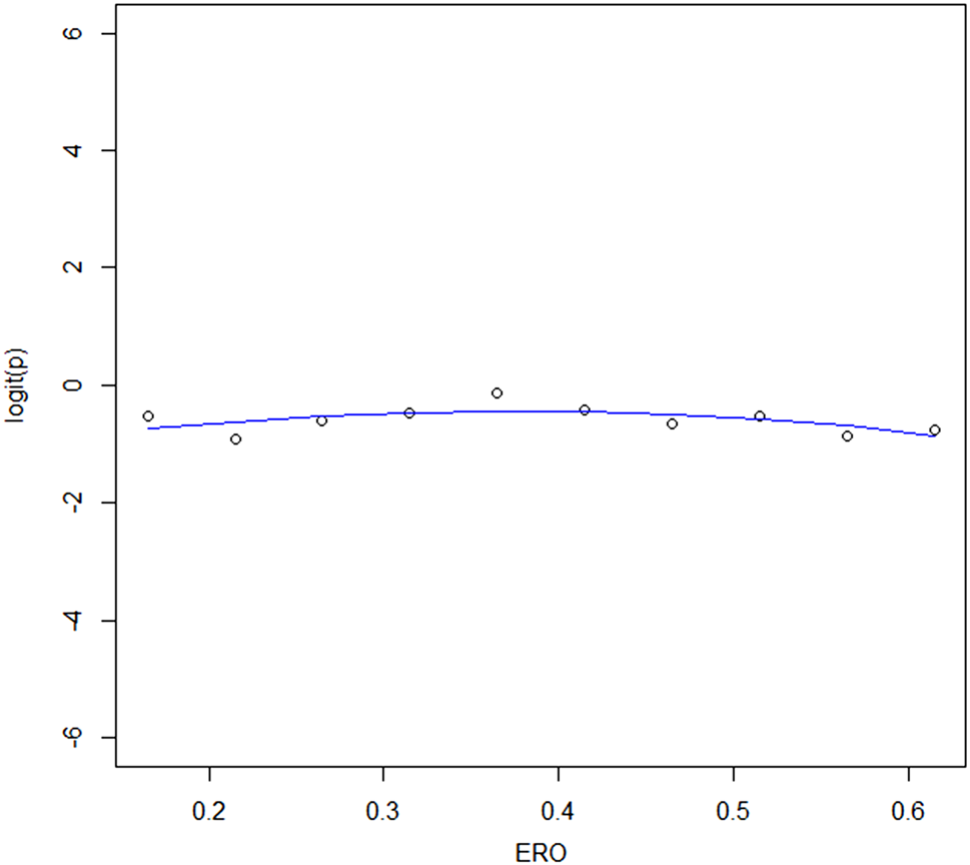
Spline curve analysis assessing the Association between ERO and mortality. Spline curve analysis revealed a flat curve, without identification of an optimal cut-off for ERO. *ERO* effective regurgitant orifice

Spline curve analysis assessing the relation between ERO and mortality revealed a flat curve, without identification of an optimal cut-off for ERO (Fig. 1). Accordingly, an ERO < and ≥ 0.3 cm^2^ was applied to evaluate the impact of ERO on mortality in this study, which is the next round value close to the median ERO of 0.28 cm^2^.

**Fig. 2:**
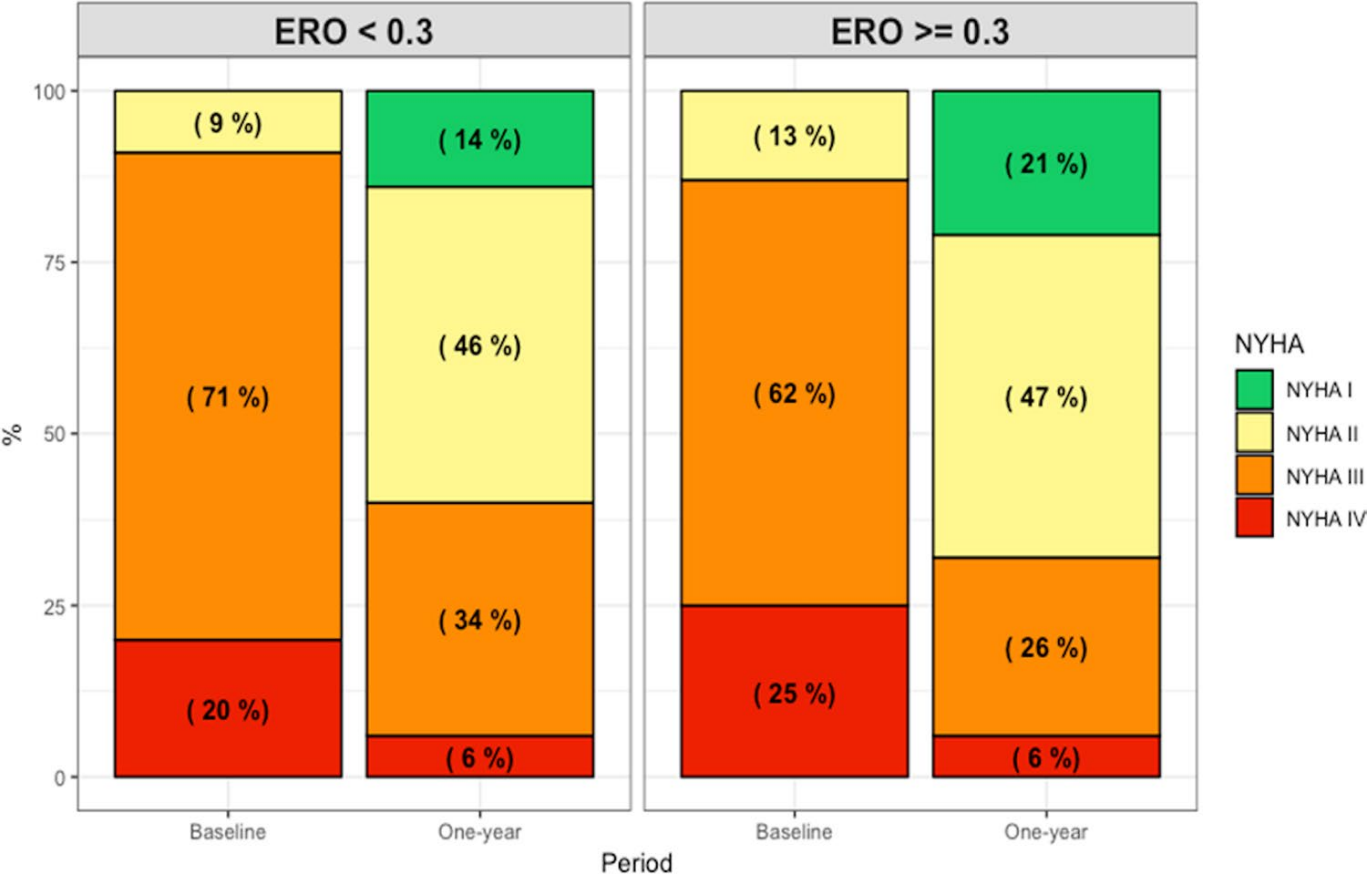
NYHA Functional Class at baseline and 1-year follow-up according to baseline ERO. Patients with baseline ERO < 0.3 cm^2^ were more frequently in NYHA class III-IV at baseline and at last follow-up, compared to patients with baseline ERO ≥ 0.3 cm^2^ (*P* = 0.002 and *P* = 0.050, respectively). *ERO* effective regurgitant orifice, *NYHA* New York Heart Association

Baseline clinical characteristics of patients according to baseline ERO are presented in Table 1. The ERO was < 0.3 cm^2^ in 575 patients (54.1%), and ≥ 0.3 cm^2^ in 487 patients (45.9%). Overall, there was no difference between the two groups except for a lower proportion of men in the lower ERO group (60.7% vs. 66.7%, for ERO < vs. ≥ 0.3 cm^2^, respectively, *P* = 0.050), and a more frequent ischemic aetiology (54.9% vs. 47.9%, *P* = 0.031). Patients in the lower ERO group were more symptomatic with a higher proportion of patients having a NYHA class of III or IV at baseline (91.4% vs. 86.9%, *P* = 0.004; Fig. 2).

Regarding baseline echocardiographic parameters, patients in the lower ERO group had smaller LV volumes with a smaller end-diastolic LV volume (166 ml vs. 174 ml, for ERO < vs. ≥ 0.3 cm^2^, respectively, *P* = 0.002). Their vena contracta diameters were smaller (6.5 mm vs. 7.0 mm, *P* < 0.018). Notably, there was no difference in LVEF between the two groups (Table 2).

### Outcome according to ERO

Post-procedural MR reduction of at least one grade was observed in 95.8% of patients (96.4% vs. 94.6%, for ERO < vs. ≥ 0.3 cm^2^, respectively, *P* = 0.530). Median MR reduction was of 2 grades [IQR 2.0–3.0] in patients with ERO < 0.3, and 2.0 [IQR: 1.5–3.0] in patients with ERO ≥ 0.3 (*P* = 0.758). This improvement was maintained at last follow-up with a median MR grade of 1 [IQR: 1.0–2.0] in the global population and in both ERO groups (*P* = 0.22).

**Fig. 3:**
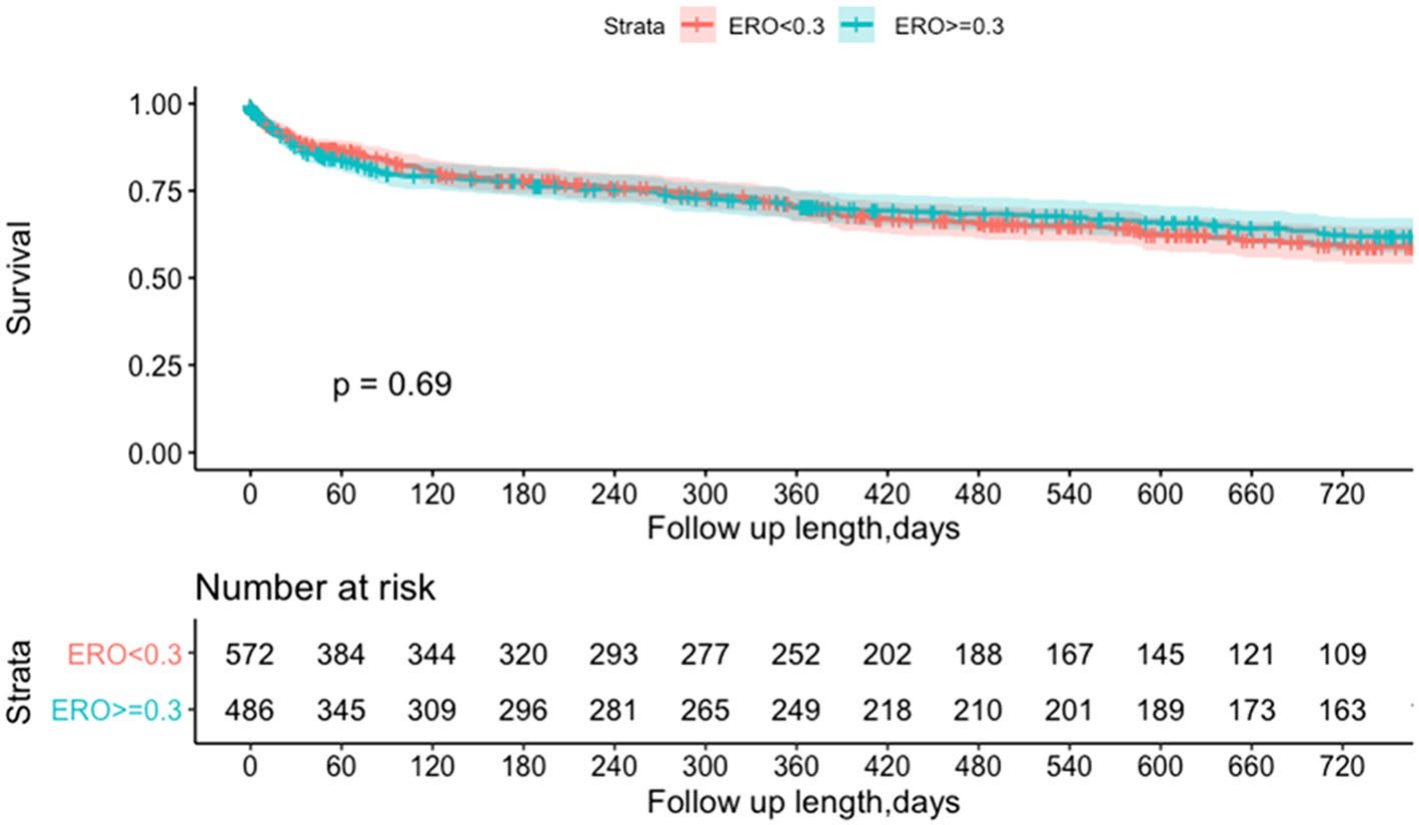
Kaplan–Meier Curve for Survival Free according to Baseline ERO. There was no difference in survival rate at 2 years according to baseline ERO (Log Rank *P* = 0.52). *ERO* effective regurgitant orifice, *TMVR* transcatheter mitral valve repair

Mean follow-up duration was 467 ± 638 days [median duration 324 (IQR: 34–730) days]. Estimated all-cause mortality rates at 1 year and 2 years were 24.0% and 29.1%, respectively. There was no difference in survival at 1 year (23.3 vs. 24.9%, *P* = 0.581) and two years (28.3% vs. 30.0%, for ERO < vs. ≥ 0.3 cm^2^, respectively, *P* = 0.585; Fig. 3). Similar results were obtained with the pre-specified cut-offs of 0.2 and 0.4 cm^2^, and with the exact median of 0.28 cm^2^ used as cut-off (Supplementary Figs. 2, 3 and 4).

At 1 year, improvement by at least one NYHA class was observed in 65.4% of patients. The improvement rate was superior in patients with a higher ERO group (61.7% vs. 73.8%, for ERO < vs. ≥ 0.3 cm^2^, respectively, *P* = 0.002). Similarly, patients in the higher ERO group were more often in NYHA class I or II (60.0% vs. 67.4%, for ERO < vs. ≥ 0.3 cm^2^, respectively, *P* = 0.05; Fig. 2). Similar results were obtained with a cut-off of 0.4cm^2^ (*P* = 0.029), while there was no difference in NYHA class at last follow-up when a cut-off of 0.2 cm^2^ was used (Supplementary Figs. 5 and 6).

## Discussion

There has been a long debate on the impact of TMVR on outcomes in patients with chronic SMR and HFrEF. While four studies, three retrospective reports and the prospective randomized COAPT trial, demonstrated a survival benefit with TMVR [6, 7, 8, 10], the prospective randomized MITRA-FR study did not [9]. These contradictory outcomes have raised a host of new questions:

1. Can the differences in outcomes between these studies be explained by differences in SMR severity as assessed by ERO?
2. Should we restrict TMVR to patients with higher ERO values?
3. Should the observed study results lead to a change of the European recommendations on the echocardiographic definition of severe SMR towards a higher ERO threshold?

The EuroSMR registry was initiated to provide further insights and answers for the raised questions. Eight European high volume centres with a long experience in the treatment of SMR by TMVR included their patients, who were treated in clinical routine, into this retrospective real-world registry. Of note, a multidisciplinary heart team assessed each individual patient before the procedure to establish the severity of MR using a multiparametric echocardiographic approach, and the appropriateness of TMVR. When compared to the COAPT-TMVR patients group, EuroSMR patients with an ERO ≥ 0.3 cm^2^ had several similarities in their baseline characteristics, including ERO (0.4 cm^2^ vs. 0.41 cm^2^, even though not all patients in COAPT did have an ERO > 0.3 cm^2^), LVEDV (174 ml vs. 194 ml), and LV-EF (33.2% vs. 31.3%) for EuroSMR vs. COAPT-TMVR, respectively. Furthermore, the two-year all-cause mortality rate was comparable with 30.0% vs. 29.1% for EuroSMR vs. COAPT-TMVR, respectively [10]. Due to the lack of a control group in EuroSMR, the true benefit of TMVR in this particular European patient population remains unclear, but the similarities with the COAPT-TMVR group might indicate a comparable outcome.

The surprising finding of EuroSMR is the comparable all-cause mortality rate in patients treated with TMVR and with an ERO < 0.3 cm^2^, without any survival difference at 1 year and 2 years. Besides, spline curves showed a very weak relation between ERO and mortality, supporting the notion that ERO may not be an appropriate tool to predict outcome after TMVR, and that differences in ERO cannot solely explain the differences in outcomes between COAPT and MITRA-FR. Accordingly, a more integrative approach, assessing among others MR severity with additional qualitative and quantitative parameters, right heart function and clinical parameters, is needed to predict which patients with HFrEF will actually benefit from TMVR, or more importantly, in which patients TMVR is ineffective.

Quantification of SMR severity is challenging, and the interpretation of the MR severity assessment should be made with caution. LV volume and stroke volume, as determined by two-dimensional echocardiography with the use of Simpson’s rule, are often underestimated, especially in patients with dilated ventricles [15, 16]. Regarding ERO, the cut-off of > 0.2 cm^2^ has traditionally been used in Europe to determine severe MR since data suggested that, adverse outcomes are associated with a smaller ERO in SMR than in primary MR. This observation is explained by a smaller regurgitant volume, which may still represent a relevant regurgitant fraction in the presence of compromised LV systolic function (and low total stroke volume) adding to the effects of elevated ventricular filling pressures. However, ERO calculated by PISA is affected by valve morphology and colour flow jet characteristics [17]. PISA measurement requires accurate visualisation of the aliasing line of the isovelocity surface and the regurgitant orifice, and suboptimal images can alter the reliability of the measurement. Besides, in SMR, Doppler methods for ERO area calculations by the flow convergence method are less reproducible and may underestimate severity because of the crescent shape of the regurgitant orifice. Finally, the reliability of ERO measurement in the setting of SMR is questionable given the variability of MR throughout systole, typically in a biphasic pattern, leading to a variation in measured ERO according to the timing of assessment, with an early systolic peak, followed by a rapid decrease to a midsystolic lowest value, then a rapid increase toward a late systolic peak [18]. The current EuroSMR findings demonstrate that selected patients with ERO above but also below 0.3 cm^2^ benefit from TMVR, and have similar mortalities, and that it might not be reasonable to restrict TMVR to patients with ERO > 0.3 cm^2^ [1, 2, 19].

Regarding the symptomatic improvement, MITRA-FR and COAPT showed a benefit of TMVR in terms of NYHA functional class. In COAPT, 72.2% of TMVR treated patients were in NYHA functional class I or II at 1-year follow-up, which compares well with the observed 68% rate of EuroSMR patients with an ERO ≥ 0.3 cm^2^. As for patients with ERO ≤ 0.3 cm^2^, the COAPT investigators recently presented the improvements in the physical exercise capacity and quality of life in patients with an ERO ≤ 0.3 cm^2^ and large left ventricles vs. the remainder of patients (either ERO > 0.3 cm^2^ or large left ventricles) [20]. In this analysis, both patient groups exhibited very similar improvements for both outcome parameters, indicating that also patients with smaller ERO experience a significant symptomatic benefit. This observation is also paralleled in the current EuroSMR registry with 61.7% of patients with an ERO < 0.3 cm^2^ demonstrating an improvement of at least one NYHA functional class at follow-up. Given this recent COAPT subgroup analysis, as well as the EuroSMR results, which both demonstrate that a large proportion of patients with severe SMR and an ERO < 0.3 cm^2^ will obtain a clinically relevant improvement from TMVR, TMVR should not be withheld from patients with an ERO < 0.3 cm^2^. However, these results should not lead to excessive TMVR in patients with ERO < 0.3cm^2^. Instead, experienced multidisciplinary heart teams have to assess TMVR appropriateness using an integrative approach of clinical aspects including the patient history of heart failure and current presentation, as well as qualitative, semi-quantitative, and quantitative echocardiographic parameters for the assessment of SMR severity [21].

## Limitations

Several limitations have to be acknowledged. This is an observational study without a control group, and there was no central adjudication of clinical status and echocardiographic parameters. Severity of MR was assessed using integrative approach including several echocardiographic parameters. Some of those variables, such as E wave velocity, pulmonary vein flow reversal, vena contracta area by 3D imaging or another measure of non-circular orifice were not consistently available in the database due to the absence of corelab and the retrospective multicentric nature of the registry that prevented the extensive collection of all echocardiographic parameters. All patients included were considered as under OMT, based on the local heart teams’ assessment; however, there was no clinical core lab to monitor this information, and the subsequent therapeutic adjustments were not documented. Several patients were lost to follow-up, as it is often the case in multicentric retrospective registries, and we do not have the specific cause of mortality, in particular, we cannot distinguish whether mortality was due to a cardiovascular cause. Finally, a selection bias might explain the observed differences in NYHA class before TMVR, e.g., the increased severity of the symptoms might explain why patients with lower ERO underwent TMVR despite a less severe MR according to ERO. However, as discussed above, it appears important to realize that the symptomatic improvement is comparable in patients with an ERO < or ≥ 0.3 cm^2^.

## Conclusion

The large EuroSMR registry reveals that ERO is not an appropriate tool to select SMR patients likely to benefit from TMVR. Besides, all-cause mortality after edge-to-edge TMVR does not differ between patients with a baseline ERO < or ≥ 0.3 cm^2^, as long as the severity of MR was established using an integrative approach, and both groups exhibited a relevant clinical improvement, so that TMVR should not be withheld from such patients who remain symptomatic despite OMT, if the appropriateness for TMVR has been determined by an experienced heart team in dedicated heart valve centres.

## Supplementary Information

Below is the link to the electronic supplementary material.

Supplementary file1 (DOCX 5230 KB)
