# Supplementary material for: Impact of effective regurgitant orifice area on outcome of secondary mitral regurgitation transcatheter repair
Source: Clin Res Cardiol. 2021 Mar 4;110(5):732–9. doi: 10.1007/s00392-021-01807-0 (PMC8099828; doi:10.1007/s00392-021-01807-0)
Supplement: Supplementary file 1 — Supplementary file1 (DOCX 5230 KB) [file 392_2021_1807_MOESM1_ESM.docx]

**Supplementary figures**

**Supplementary Figure 1. Proximal Isovelocity Surface Area (PISA) radius measurement**


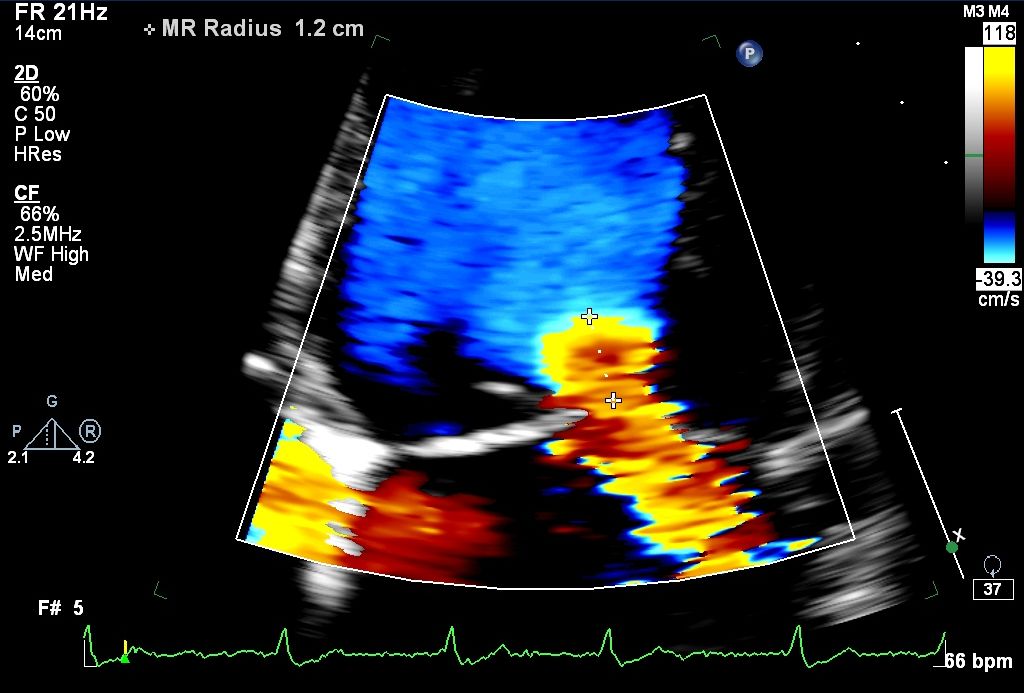


**Aliasing line**

**PISA radius**

**Regurgitant orifice**

**Left atrium**

**Left ventricle**

**Supplementary Figure 2.** Kaplan Meyer Curve for Survival Free according to Baseline ERO (ERO cut-off 0.4cm^2^).


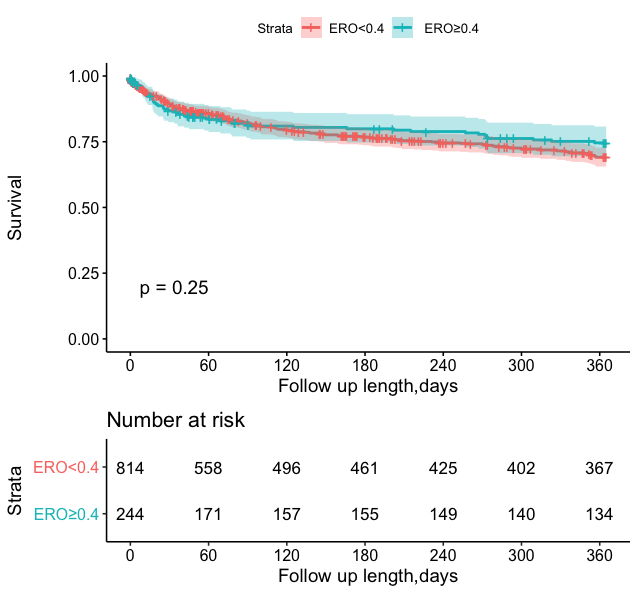


ERO: effective regurgitant orifice

**Supplementary Figure 3.** Kaplan Meyer Curve for Survival Free according to Baseline ERO (ERO cut-off 0.2cm^2^).


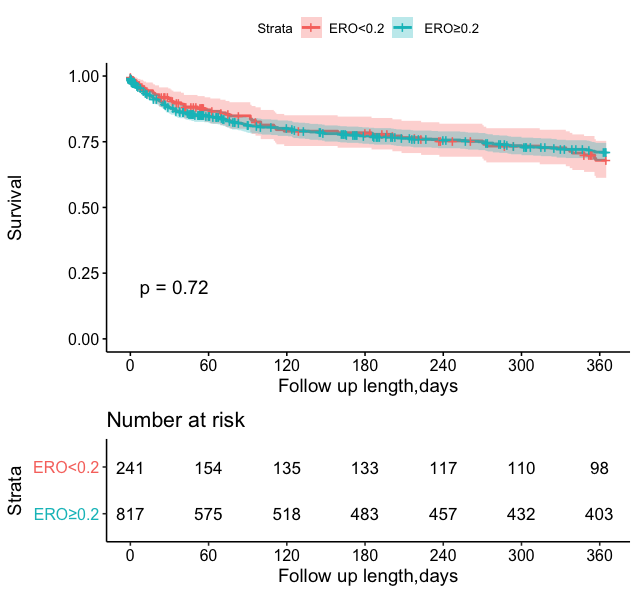


ERO: effective regurgitant orifice

**Figure 4.** Kaplan Meyer Curve for Survival Free according to Baseline ERO with the median used as cut-off.


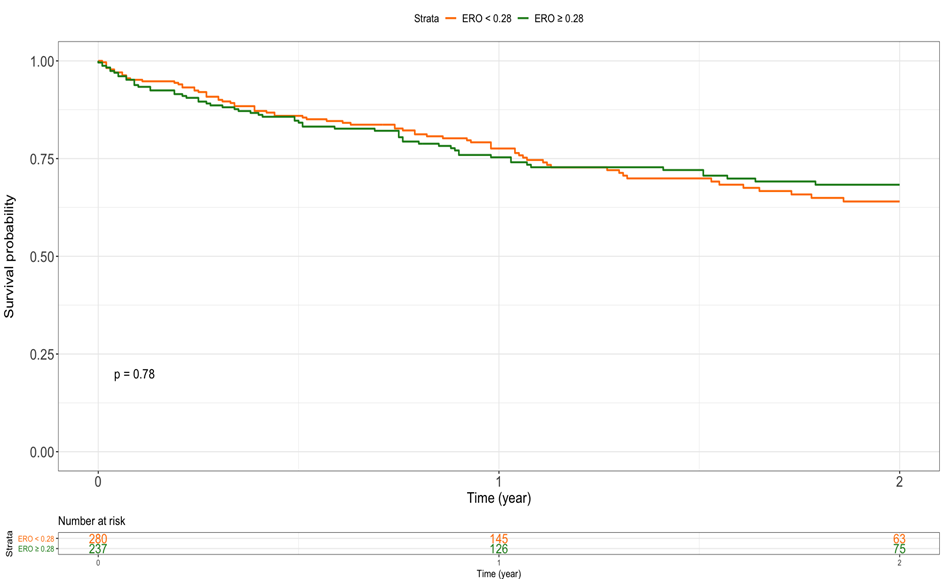


ERO: effective regurgitant orifice

**Supplementary Figure 2.** NYHA Functional Class at Baseline and 1-year Follow-up According to Baseline ERO (ERO cut-off 0.4cm^2^).


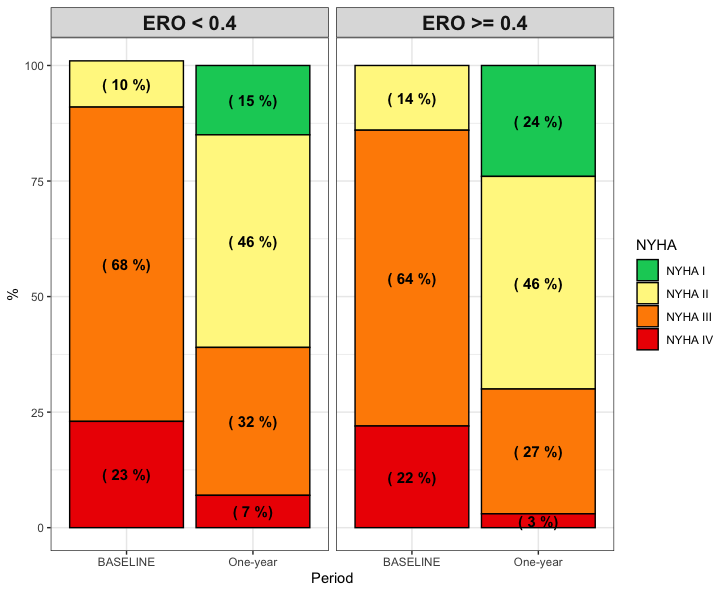


There was no difference in baseline NYHA class between the 2 ERO groups (P= 0.19). At last follow-up, patients in the higher ERO group were more frequently in NYHA class I or II (P=0.029). ERO: effective regurgitant orifice; NYHA: New York Heart Association

**Supplementary Figure 3.** NYHA Functional Class at Baseline and 1-year Follow-up According to Baseline ERO (ERO cut-off 0.2cm^2^).


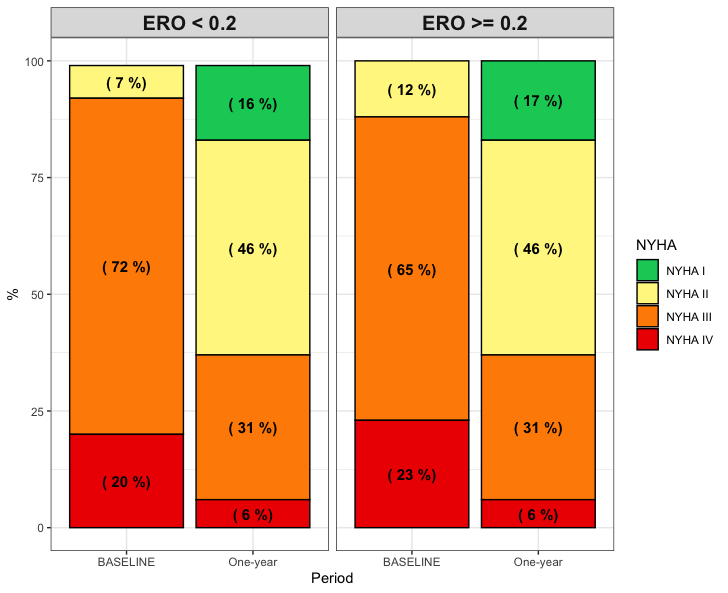


There was a trend for more severe NYHA class in patients in the lower ERO group at baseline (P=0.007), while at last follow-up, there was no difference in NYHA between the 2 groups (P=0.98). ERO: effective regurgitant orifice; NYHA: New York Heart Association
